# Epidemiology of TB in the hot zone of Sudan’s armed conflict following 3 years of catastrophic war

**DOI:** 10.5588/ijtldopen.26.0351

**Published:** 2026-11-13

**Authors:** A.K.Y. Mohammed, E.H.M. Humida, N.A. Hamid, H.G. Ahmed

**Affiliations:** 1 Department of Medicine, Faculty of Medicine, University of Kordofan, El-Obeid, Sudan;; 2 El-Obeid International Hospital, Aldaman, El-Obeid, Sudan;; 3 EL-Obeid Teaching Hospital, El-Obeid, Sudan;; 4 EL-Obeid International Hospital–Cardiac Catheterization Laboratory, EL-Obeid, Sudan;; 5 Prof Medical Research Consultancy Center, El-Obeid, Sudan;; 6 Department of Histopathology and Cytology, FMLS, University of Khartoum, Khartoum, Sudan.

**Keywords:** tuberculosis, pulmonary TB, extra-pulmonary TB, Kordofan, Sudan

## Abstract

**BACKGROUND:**

TB is a major global health problem, particularly in low-resource, armed conflict areas. This study aimed to assess the epidemiology of TB in a hot conflict zone after 3 years of continuing armed conflict in western Sudan.

**METHODOLOGY:**

This is a retrospective descriptive study conducted at El-Obeid Teaching Hospital in North Kordofan State, Sudan, from April 2023 to April 2026. We obtained all data on patients diagnosed with TB from the hospital. The sample size includes all TB patients throughout the designated period. About 3,498 people displayed signs of TB, of whom 2,193 got a confirmed diagnosis.

**RESULTS:**

The overall prevalence of TB was 85 cases per 100,000 people. The prevalence of pulmonary TB was 57 per 100,000, and extra-pulmonary TB was 28 per 100,000 individuals. Men represented 70.2%, and women were 29.8%. The 21–29 age group had the highest prevalence, followed by 30–39, 40–54, 55+, and ≤20 years, with rates of 22.5%, 20.8%, 20.7%, 19.2%, and 16.6%.

**CONCLUSION:**

There is an increase in the prevalence of TB in Western Sudan, particularly among younger adult men and gold mining workers. This data indicates that TB is a major public health issue in the region, particularly among at-risk populations.

TB continues to pose a substantial public health challenge worldwide, especially in areas experiencing humanitarian crises and conflict.^1^ Sudan is experiencing a prolonged, large-scale humanitarian emergency in which conflict, displacement, and the fragmentation of health services have become key determinants of infectious-disease risk. The crisis has displaced around 15.8 million people, causing catastrophic levels of acute food insecurity and systemic health collapse.^2^ Epidemiological conditions, including population movements, overcrowding in camps and host communities, and interruptions in preventive and diagnostic care, create an environment conducive to TB transmission and delays in detection and treatment. In conflict-affected areas, disruption of routine primary health care, reduced availability of TB diagnostic and drug supplies, damaged or inaccessible facilities, staff shortages, and barriers to referral and follow-up further weaken TB control and continuity of care.^3^ This context is especially relevant for conflict-linked ‘hot zones’, where health system instability and civilian vulnerability intersect, amplifying both TB incidence and the likelihood of ongoing transmission and adverse outcomes.

Sudan has experienced prolonged periods of conflict and social instability over the decades, exemplifying this trend. The ongoing conflict, intensified by economic instability and health care service disruptions, has led to a precarious epidemiological situation for infectious diseases, such as TB.^4^ It is anticipated that TB accounted for 1% of total inpatient mortality in Sudan in 2017.^5^ A recent study found that 71% of TB patients had pulmonary disease and 29% had extra-pulmonary disease. About 74.3% of pulmonary TB patients were men, and 25.7% were women.^6^ The Sudanese civil war increased TB incidence from 54 to 63 per 100,000. Multidrug resistance, recurrent disease, and noncompliance increased during conflict. Men, labourers, and gold miners are particularly vulnerable to the disease. Lymph nodes, pleura, and abdomen increasingly exhibit extra-pulmonary TB. Sudan needs immediate improvements to its health care system to stop TB transmission.^7,8^ Understanding Sudan’s TB epidemiology after 3 years of war is essential for public health interventions and resource allocation. The conflict’s social and infrastructural disturbances may have changed TB transmission patterns, affecting incidence, prevalence, and demographics. TB epidemiology must also be examined in light of restricted health care access, drug shortages, and public health infrastructure decline.

This study investigated the epidemiology of TB in Sudan after 3 years of armed conflict. Identifying epidemiologic metrics such as prevalence, demographics, war-related factors, and outcomes can guide future public health strategies and actions for managing and combating TB in Sudan, while also contributing to a wider discourse on conflict and infectious-disease epidemiology. This study emphasises the necessity of specific public health interventions in a region that is still in the process of recovery from conflict and instability.

## METHODS

This is a retrospective descriptive study conducted at El-Obeid Teaching Hospital in North Kordofan State, Sudan, from April 2023 to April 2026. North Kordofan, a state in Sudan with a population of about 2,577,000, is made up of both sedentary farmers and pastoralist nomads. The population is predominantly rural (67%), with approximately 20% urban and 13% nomadic groups. North Kordofan emerged as the principal destination, accommodating 25% of the displaced people^9,10^ (see Figure). We obtained all data on patients diagnosed with TB from the hospital. The sample size included the entire population of TB patients throughout the designated period (3 years during the Sudan War, 2023–2026). During the period, about 3,498 people displayed signs of TB, with 2,193 acquiring a confirmed diagnosis. The patient files contain not only clinical information but also demographic data, including age, sex, education level, occupation, marital status, and residence.

**Figure.**
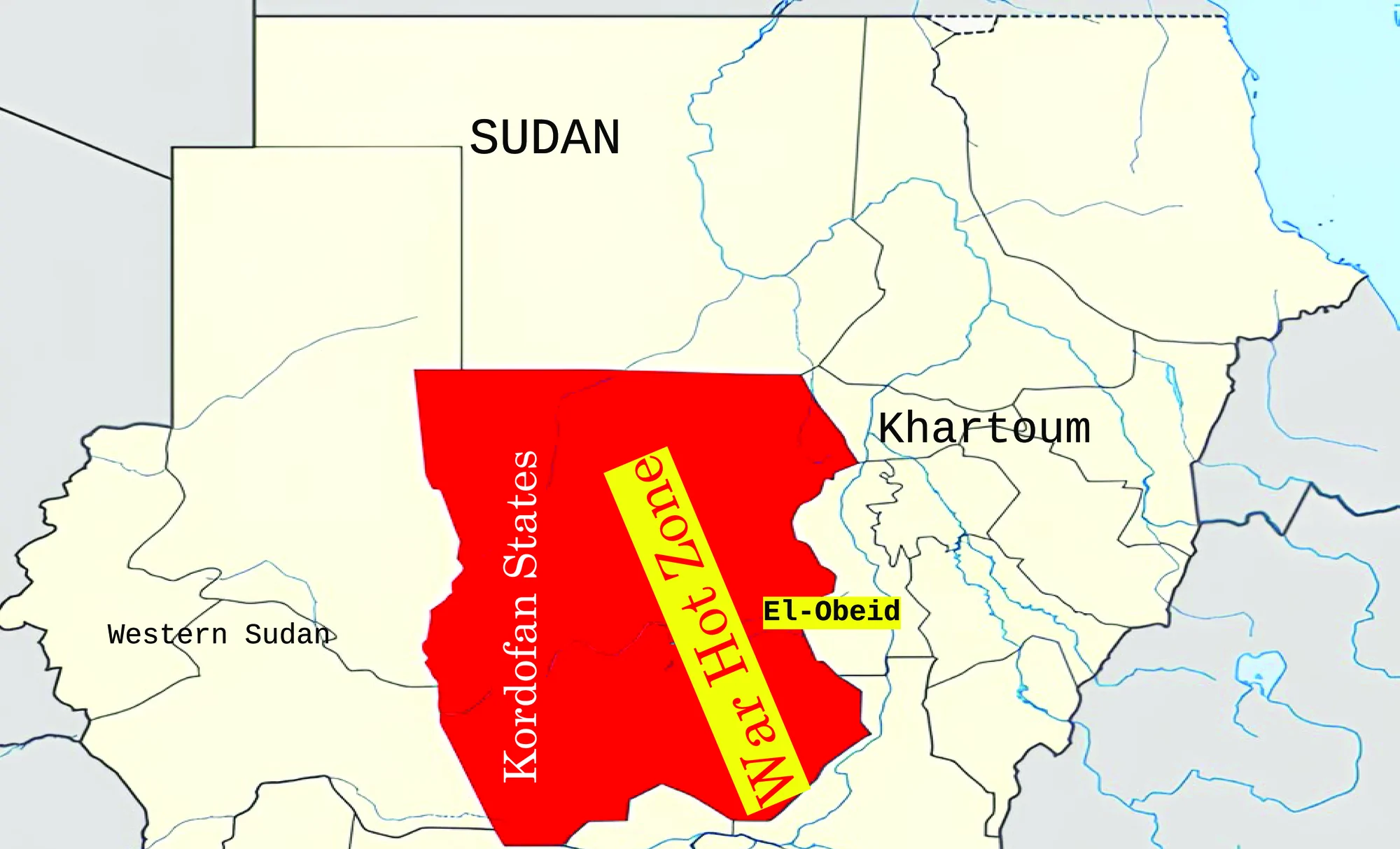
Map of Sudan showing war hot zone areas, as of 2026.

The diagnosis of TB relies on medical history, physical examination, TB blood or skin tests, chest radiography, and bacteriological assessment (including sputum smear microscopy, nucleic acid amplification testing, culture, and drug susceptibility testing).

### Data analysis

All collected data were systematically organised into data sheets and subsequently entered into the Statistical Package for Social Sciences (SPSS) version 31, based in Chicago, USA. We analysed the data to obtain frequencies, percentages, relative risk (RR), and cross-tabulations. The calculation of the χ² test was performed at a 95% confidence level. *P* values less than 0.05 were deemed statistically significant.

### Ethical statement

The study was approved by the Prof Medical Research Consultancy Center’s Institutional Review Board (IRB) with the reference number HREC 0014/PMRCC.9/24. The study followed the Helsinki declaration, and all participants provided signed informed consent before data collection.

## RESULTS

This study investigated 2193 TB patients aged 1–105 years, with a mean age of 37 years. The overall prevalence of TB in North Kordofan State was 85 per 100,000 individuals. The prevalence of pulmonary TB was 57 per 100,000, and extra-pulmonary TB was 28 per 100,000 people.

Of the 1,539 patients, 70.2% were men and 29.8% were women. The highest infected age group was 21–29 years, followed by 30–39, representing 22.5% and 20.8%, respectively. Urban residents represented 52.2% compared to 47.8% of TB patients in rural areas. Most patients had a basic education level (69.5%). The highest TB infection was seen among gold mining workers, followed by labourers, constituting 28.8% and 20.2%, in that order, as indicated in Table 1. Out of TB patients, 1,463/2,193 (66.7%) were pulmonary TB and 730/2,193 (33.3%) were extra-pulmonary TB patients. Pulmonary TB among men was 70.8% and 57.2% in women. The risk of pulmonary TB among men was as follows: RR (95% confidence interval [CI]) = 1.2374 (1.1495–1.3320), *P* < 0.0001.

**Table 1.** Distribution of the patients by their demographic characteristics.

Variable	Men, n = 1,539	Women, n = 654	Total, n = 2,193
Age			
≤20 years	221 (14.4%)	143 (21.9%)	364 (16.6%)
21–29	378 (24.6%)	116 (17.7%)	494 (22.5%)
30–39	338 (22%)	119 (18.2%)	457 (20.%)
40–54	322 (21%)	134 (20.5%)	456 (20.7%)
55+	280 (18.2%)	142 (21.7%)	422 (19.2%)
Residences			
Urban	760 (49.4%)	391 (59.8%)	1,151 (52.5%)
Rural	779 (50.6%)	263 (40.2%)	1,042 (47.5%)
Education			
Illiterate	280 (18.2%)	163 (24.9%)	443 (20.2%)
Basic	1,096 (71.2%)	428 (65.4%)	1,524 (69.5%)
University	163 (10.6%)	63 (9.6%)	226 (10.3%)
Occupation			
Gold mining	607 (39.4%)	24 (3.7%)	631 (28.8%)
Farmers	178 (11.6%)	34 (5.2%)	212 (9.7%)
Labourers	423 (27.5%)	19 (2.9%)	442 (20.2%)
Students	86 (5.6%)	120 (18.3%)	206 (9.4%)
Medical	9 (0.6%)	9 (1.4%)	18 (0.8%)
Housewife	9 (0.6%)	387 (59.2%)	396 (18%)
Others	227 (14.7%)	61 (9.3%)	288 (13%)
Marital status			
Single	653 (42.4%)	193 (29.5%)	846 (38.6%)
Married	876 (57%)	431 (66%)	1,307 (59.6%)
Divorce	3 (0.2%)	5 (0.8%)	8 (0.4%)
Widow	6 (0.4%)	22 (3.4%)	28 (1.3%)
Engaged	1 (0.06%)	3 (0.5%)	4 (0.2%)

Extra-pulmonary TB among men was 29.2% and 42.8% in women. The risk of extra-pulmonary TB among women was as follows: RR (95% CI) = 0.8082 (0.7508–0.87), *P* < 0.01, z-statistics = 5.665.

The highest pulmonary TB infection was observed in the age range 21–29 years, followed by 30–39 and 40–54, representing 26.2% and 22.4%. The highest extra-pulmonary TB infection was observed in individuals aged 20 years and younger, followed by those aged 40–54, representing 26.4% and 21.4%, respectively. The risk of pulmonary TB among those 30 years or older was as follows: RR (95% CI) = 1.492 (1.3571–1.6403), *P* < 0.0001, and z-statistic = 8.278. The risk of extra-pulmonary TB among those under 30 years was as follows: RR (95% CI) = 0.6702 (0.6097–0.7368), *P* < 0.001, z-statistic = 8.278.

Pulmonary TB was higher among rural residents (51.7% vs. 48.3% urban residents), whereas extra-pulmonary TB was higher among urban residents (60.8% vs. rural residents 39.2%). The risk of extra-pulmonary TB among urban residents was as follows: RR (95% CI) = 1.2586 (1.1633–1.3617), *P* < 0.0001, z-statistics = 5.726. The highest pulmonary TB infection was among gold mining workers, followed by labourers, constituting 36.4% and 20%, in this order. The highest extra-pulmonary TB infection was among both labourers and housewives (20.5% each), followed by students, representing 15.2% and 13.6%, respectively, as indicated in Table 2.

**Table 2.** Distribution of the TB type by demographic features.

Variable	Pulmonary TB, n = 1,463	Extra-pulmonary TB, n = 730	Total, n = 2,193
Sex			
Male	1,089 (74.4%)	450 (61.6%)	1,539 (70.2%)
Female	374 (25.6%)	280 (38.4%)	654 (29.8%)
Marital status			
Single	543 (37%)	303 (41.5%)	846 (38.6%)
Married	891 (60.9%)	416 (57%)	1,307 (59.6%)
Divorced	5 (0.3%)	3 (0.4%)	8 (0.4%)
Widow	21 (1.4%)	7 (1%)	28 (1.3%)
Engaged	3 (0.2%)	1 (0.14%)	4 (0.2%)
Age			
≤20 years	171 (11.7%)	193 (26.4%)	364 (16.6%)
21–29	383 (26.2%)	111 (15.2%)	494 (4.3%)
30–39	328 (22.4%)	129 (17.7%)	457 (20.8%)
40–54	300 (20.5%)	156 (2%)	456 (20.7%)
55+	281 (19.2%)	141 (19.3%)	422 (19.2%)
Residence			
Urban	707 (48.3%)	444 (60.8%)	1,151 (52.5%)
Rural	756 (51.7%)	286 (39.2%)	1,042 (47.5%)
Education			
illiterate	283 (19.3%)	160 (22%)	443 (20.2%)
Basic	1,033 (70.6%)	491 (67.2%)	1,524 (69.5%)
University	147 (10%)	79 (10.8%)	226 (10.3%)
Occupation			
Gold mining	532 (36.4%)	99 (13.6%)	631 (28.8%)
Farmers	146 (10%)	66 (9%)	212 (9.7%)
Labourers	292 (20%)	150 (20.5%)	442 (20.2%)
Students	95 (6.5%)	111 (15.2%)	206 (9.4%)
Medicals	7 (0.5%)	11 (1.5%)	18 (0.8%)
Housewives	246 (16.8%)	150 (20.5%)	396 (18%)
Others	145 (9.8%)	143 (19.6%)	288 (13%)

Regarding the outcomes, the overall mortality was 141/2,193 (6.4%), of whom 83/1,463 (5.7%) were among pulmonary TB cases, and 58/730 (8%) were among extra-pulmonary TB cases. Of 188/2,193 (8.6%) defaulted patients, 126/1,463 (8.6%) had pulmonary TB and 62/730 (8.5%) had extra-pulmonary TB, as indicated in Table 3.

**Table 3.** Distribution of the TB type by outcomes.

Outcomes	Pulmonary TB	Extra-pulmonary TB	Total
Cured	299	26	325
Completed	518	360	878
Transferred	5	8	13
Defaulted	126	62	188
Failed	1	0	1
Died	83	58	141
In medication	431	216	647

## DISCUSSION

The reported prevalence rates of TB in the present study, whether they be total (85 per 100,000), pulmonary (57 per 100,000), or extra-pulmonary TB (28 per 100,000), are very high compared to the previous ones from Sudan. They are a cause for serious public health concern and should be thoroughly investigated. These rates of occurrence highlight the difficulties encountered in the area, particularly following the war and the continuing socio-economic crises. These prevalence rates are the highest ever compared to the previously reported rates, which stand at 58 per 100,000 persons.^3,5^ These high prevalence rates can be explained by several factors, all of which are critical to comprehending the TB situation in the North Kordofan state of Sudan.

Morocco has the highest TB rate in North Africa, at 98 cases per 100,000, followed by Libya (63 per 100,000) and Algeria (54 per 100,000), according to prior reports. North African countries (including Sudan) have some of the lowest TB rates in Africa compared to sub-Saharan Africa.^11^ Ethiopia, which shares a border with Sudan, is experiencing significant internal displacement. As of February 2022, there were 4.56 million internally displaced individuals, with 3.85 million (84%) displaced due to conflict. This population has limited access to facility-based TB care, and the increasing number of untreated TB cases among the displaced poses a risk for community transmission. Of the 199,398 individuals from conflict-affected populations screened for TB, 10,585 (5%) were identified as presumptive cases, and 353 (2%) were bacteriologically confirmed cases of pulmonary TB that were diagnosed and treated. The incidence of TB among the conflict-affected population (1,126 cases) and prison inmates (1,111 cases) is nine times higher than the national annual TB incidence estimate of 126 per 100,000. The elevated TB rates in conflict-affected groups demonstrate the critical need for targeted interventions.^12^

This study found that the prevalence of TB was significantly greater in men compared to women. The observed male predominance may indicate variations in exposure to TB risk factors and health-seeking behaviours. Men may experience greater exposure due to occupational and social activities; potentially elevated rates of smoking and co-morbidities; as well as delayed presentation to care in certain contexts, all of which can lead to a higher diagnosed and reported burden of TB among men. Gender disparities in TB incidence have been documented globally. The global incidence of TB is markedly higher in men compared to women, with men representing approximately 54%–56% of cases. Research indicates that men experience a TB incidence rate 1.7 times greater than that of women. Elevated levels of smoking, alcohol use, and greater social, occupational, or environmental exposure, especially among adults aged 15 and older, contribute to this disparity. Various factors have been suggested to elucidate the gender gap, including biological differences in disease and its presentation, as well as disparities in access to health care, particularly in developing countries.^13^

The age distribution exhibited a non-uniform pattern in the present study. The highest prevalence of TB was observed in the 21–29 age group (22.5%), followed by the 30–39 age group (20.8%) and the 40–54 age group (20.7%). The prevalence was lower in individuals aged 55 years and older (19.2%) and those aged 20 years and younger (16.6%). The findings indicate that the burden of TB is highest among young and middle-aged adults, a demographic typically associated with increased social interaction, workforce engagement, and, depending on the local context, elevated levels of crowding or delayed diagnosis. The lower prevalence observed in younger adolescents and children may suggest variations in susceptibility, differences in symptom recognition, or obstacles to detection within this demographic. Age significantly affects the risk of TB, with adults aged 25–54 generally experiencing the highest incidence. However, there is a notable increase in risk among elderly people over 65, attributed to immunological decline and the presence of coexisting illnesses. Children under 5 years of age exhibit significant susceptibility to accelerated disease progression following infection.^14–16^

The present study also reported residence as a factor. The prevalence of TB was greater in urban populations than in rural populations. Urban predominance can be attributed to elevated population density, enhanced transmission opportunities, and a higher probability of health facility utilisation and diagnosis relative to rural areas. Alternatively, rural underdiagnosis remains a possibility, as restricted access to diagnostic services, health care expenses, and the extent of health system coverage may affect the proportion of identified TB cases. According to data projected for 2026, the assertion that TB prevalence is higher in urban populations compared to rural ones holds true on a global level, though there are notable regional variations and a trend towards the increasing urbanisation of the disease.^17^

TB prevalence varied by job type in this study, with gold mine workers having the highest frequency, followed by labourers, housewives, farmers, and students. Gold mining workers had 28.8% of TB cases, labourers 20.2%, housewives 18.1%, farmers 9.7%, and students 9.4%. These results point out the importance of occupational groups, especially gold mining workers and labourers, as priority populations for TB control. Targeted interventions may encompass enhanced screening and early diagnosis in workplaces, improved ventilation and infection control protocols, fortified referral and follow-up systems, and community-based strategies that tackle household transmission and obstacles encountered by non-working or home-based demographics, such as housewives.

This study revealed a substantial differentiation between mortality and treatment non-completion (default) in TB outcomes, with both evident in pulmonary and extra-pulmonary disease types. The overall mortality rate was 6.4%. TB site-specific mortality was 5.7% for pulmonary TB and 8.0% for extra-pulmonary TB. This shows that delayed diagnosis, more severe disease presentation, greater diagnostic difficulty, or a higher chance of underlying co-morbidities and sequelae may lower survival in extra-pulmonary TB. Extra-pulmonary illness has lengthier and more complicated diagnostic and therapeutic routes, which may worsen results.

Also significant was the default (treatment interruption). 8.6% of patients defaulted, indicating treatment continuation issues. Both pulmonary and extra-pulmonary TB had 8.6% and 8.5% default rates. This similar default rate suggests that pulmonary and extra-pulmonary TB patients face similar barriers to treatment interruption, such as limited access to follow-up services, financial constraints, war circumstances, treatment side effects, social and family responsibilities, or health facility distance.

These findings highlight two key issues for TB control: 1) the higher mortality in extra-pulmonary TB, reinforcing the need for early identification, timely treatment, and close clinical monitoring for these patients; and 2) the consistent default rate across both forms, suggesting that interventions should broadly target adherence and retention in care.

Countries under conflict, such as Myanmar, Palestine, Sudan, and Ukraine, violate human rights and international law. In Gaza, ongoing bombings have endangered respiratory health, resulted in the deaths of more than 1,500 health workers, led to the arrest of additional personnel, and caused significant damage to hospitals and ambulances. We believe that situation-specific advocacy is necessary for urgent, life-saving situations. At the 2025 Union World Conference on Lung Health in Copenhagen, the Union Board of Directors called on all parties involved, whether directly or indirectly, to make sure that international laws are followed, including protecting civilians and health care workers, ensuring access to food, sanitation, and medical supplies, and rebuilding health care systems.^18^

The study does have certain limitations. The data collection period occurred during a continuing war in Sudan, which likely impacted health care access, patient behaviour, and the overall capacity to diagnose and treat TB effectively. Disruptions to health care services during conflict can lead to variations in reported cases, affecting the reliability of prevalence estimates. Although the study spans 3 years, the conflict may change external factors affecting TB transmission dynamics. Population displacement, health care access, and socio-economic factors may affect TB incidence over time, making it difficult to study.

## CONCLUSION

Our study reveals a notable burden of TB within the population of North Kordofan State, indicating an overall prevalence rate of 85 cases per 100,000 individuals. Pulmonary TB represents a significant majority at 57 per 100,000 individuals, whereas the prevalence of extra-pulmonary TB is recorded at 28 per 100,000. This data shows that TB is a significant public health concern in the region, especially among at-risk populations. The study emphasises the necessity of improved TB control measures suited to North Kordofan’s demographic and occupational factors. To tackle the TB pandemic in the region, public health measures should emphasise awareness, prevention, and treatment adherence, especially among high-risk groups like miners and urbanites. Addressing these factors is essential for reducing TB and enhancing health in North Kordofan State.
